# P-2322. Screening Rates and Clinical Outcomes for *Hepatitis Delta* in Individuals with HBV Coinfection

**DOI:** 10.1093/ofid/ofae631.2474

**Published:** 2025-01-29

**Authors:** Adeel A Butt, Peng Yan, Obaid Shaikh, Roger Bedimo

**Affiliations:** Weill Cornell Medicine, Doha, Ad Dawhah, Qatar; VA Pittsburgh Healthcare System, Pittsburgh, Pennsylvania; VA Pittsburgh Healthcare System, Pittsburgh, Pennsylvania; VA North Texas Health Care System, Dallas, TX

## Abstract

**Background:**

Hepatitis Delta (HDV) screening is recommended for those with hepatitis B virus (HBV) infection. However, screening rates are not well documented and are far from ideal. Factors associated with screening for HDV, and clinical outcomes among HBV/HDV coinfected individuals are poorly understood. Our aim was to define the screening rates for HDV among HBV infected individuals, and the incidence of various complications among HBV/HDV coinfected individuals in the US VA healthcare system.

Incidence rates for cirrhosis, hepatic decompensation, and hepatocellular carcinoma among those with HBV and HBV/HDV coinfection.

**Figure:**
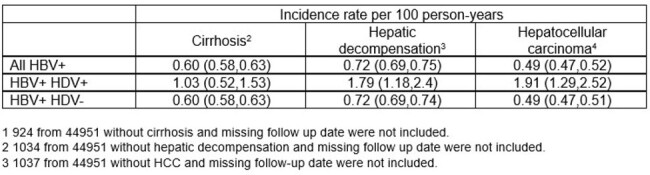

**Methods:**

Within the Veterans Health Administration (VA) national databases, we identified all individuals with HBV (defined as a positive test for HBV surface or “e” antigen, or HBV DNA) between 2002-2023. Screening for HDV was ascertained by serology or HDV PCR testing. Incidence rates for development of cirrhosis, hepatic decompensation (HD), hepatocellular carcinoma (HCC), and death were calculated for those with HBV alone and those with HBV/HDV coinfection.

Kaplan-Meier curves demonstrating progression to cirrhosis, Hepatitis D, and Hepatocellular Carcinoma

**Figure:**
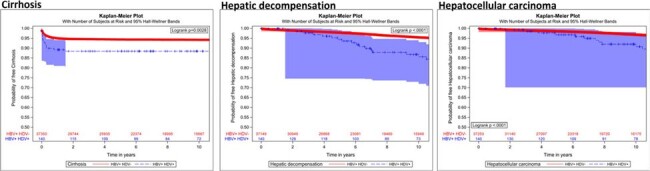

**Results:**

Of the 44,951 individuals with HBV, 5,964 (13.3%) were screened for HDV and 180 (3% of those tested) were positive for HDV. Among 38,472 individuals without baseline cirrhosis, the incidence rates/100 person years (95% CI) for cirrhosis, HD, and HCC were 1.03 (0.52,1.53), 1.79 (1.18,2.4), and 1.91 (1.29,2.52) respectively among those with HBV/HDV coinfection compared with 0.60 (0.58,0.63), 0.72 (0.69,0.74), and 0.49 (0.47,0.51) among those with HBV infection alone. Kaplan-Meier curves demonstrated a higher proportion and accelerated progression to cirrhosis, HD, and HCC among HBV/HDV coinfected compared with HBV monoinfected individuals.

**Conclusion:**

There are significant gaps in screening for HDV among HBV infected individuals. Those with HBV/HDV coinfection are more likely to develop serious complications compared with those with HBV alone.

**Disclosures:**

Adeel A. Butt, MD, MS, Gilead Sciences: Grant/Research Support

